# Determinants of the continuum of maternal health care in Cambodia: an analysis of the Cambodia demographic health survey 2014

**DOI:** 10.1186/s12884-021-03890-7

**Published:** 2021-06-02

**Authors:** Savina Chham, Emma Radovich, Veerle Buffel, Por Ir, Edwin Wouters

**Affiliations:** 1 National Institute of Public Health, Lot 80, Street 566 & Corner with 289, St 566, Phnom Penh, Cambodia; 2 London School of Hygiene and Tropical Medicine, London, UK; 3 University of Antwerp, Antwerp, Belgium

**Keywords:** Maternal Health Care, Optimal, Continuum of Care, Determinant, Dropping Out, Demographic Health Survey, Cambodia

## Abstract

**Introduction:**

Cambodia has achieved significant progress in maternal health, yet remains in the group of countries with the highest maternal mortality ratio in South-East Asia. Extra efforts are needed to improve maternal health through assessing the coverage of maternal health services as a continuum of care (CoC) and identifying the gaps. Our study aims to explore the coverage level of the Optimal CoC by (1) measuring the continuity of optimal antenatal care (ANC), skilled birth attendance (SBA) and optimal post-natal care (PNC), (2) identifying the determinants of dropping out from one service to another and (3) of not achieving the complete CoC.

**Method:**

The study employed data from the Cambodia Demographic Health Survey 2014. We restricted our analysis to married women who had a live birth in the five years preceding the survey (*n* = 5678). Bi-variate and multivariate logistic regression were performed using STATA version 14.

**Results:**

Almost 50% of women had achieved the complete optimal CoC, while the remaining have used only one or two of the services. The result shows that the level of women’s education was positively associated with the use of optimal ANC, the continuation to using optimal PNC and achieving the complete CoC. More power of women in household decision making was also positively associated with receiving the complete CoC. The birth order was negatively associated with achieving the complete CoC, while exposure to the mass media and having health insurance increased the odds of achieving the complete CoC. Household wealth consequently emerged as an influential predictor of dropping out and not achieving the complete CoC. Receiving all different elements of ANC care improved the continuity of care from optimal ANC to SBA and from SBA to optimal ANC.

**Conclusion:**

The findings urge policy makers to approach maternal health care as a continuum of care with different determinants at each step. Household wealth was found to be the most influential factor, yet the study discovered also other barriers to optimal maternal health care which need to be addressed: future intervention should thus not only aim to increase wealth or health insurance coverage but also stimulate the education of women and empower women to claim power in household decision-making.

## Introduction

Cambodia has achieved significant progress in the area of maternal and child health, meeting Millennium Development Goal 4 (MDG) to reduce child mortality and MDG5a to reduce maternal mortality [1]. Based on the 2014 Cambodia Demographic Health Survey (CDHS), under-five mortality has decreased from 124 deaths per 1000 live births in 2000 to 35 deaths per 1000 live births in 2014 [2]. Within this period, there was also a decline in maternal mortality from 206 to 170 deaths per 100 000 live births. However, Cambodia remains in the group of countries with the highest maternal mortality ratio in South-East Asia [3]. Therefore, extra effort is needed to achieve the new health target of Sustainable Development Goal (SDG 3.1) to lower the maternal mortality ratio to less than 70 per 100 000 live births by 2030 [4].

To achieve this target effectively, adequate and high-quality care for maternal health (MH) is required along the continuum of care (CoC). The coverage of MH services is regarded as one of the main contributions to the reductions of maternal mortalities [5].

Previous studies have largely focused on individual components along the continuum of care separately, especially Antenatal Care (ANC) [6–8] and to Skilled Birth Attendant (SBA) [9, 10], but that Post-natal Care (PNC) has received less attention until recently [11]. Coverage along each component can be measured by counting ANC which pregnant women should have adequate and high-quality ANC during pregnancy with at least optimum four visits as recommended by World Health Organization (WHO) [12] or eight visits following the new WHO 2016 guideline [13]. Women should have SBA which refers to delivery care provided by qualified and experienced health professional (a midwife, doctor or nurse) [14, 15]. After delivery, PNC, especially within the first 48 h after birth, is critical to the management of postpartum complications [16].

However, targeting the coverage of MH services separately does not ensure that every woman receives all essential MH services throughout pregnancy, childbirth and postpartum periods [17]. There is an increasing focus on applying a CoC approach, which indicates the continuation of care throughout the lifecycle and implies that MH services are closely linked and action must be taken in an integrated manner [18]. The CoC is widely divided into two key dimensions: time and place. The time dimension addresses the situation where women receive MH services from pre-pregnancy to post-delivery and it addresses the importance of continuity of packages of MH service over time at different stages of pregnancy, childbirth and postpartum [18–20]. The place dimension measures various components of services provided at health facilities, communities and home and addresses the coordination among family level, community-level and health facility-level [18, 20].

In this paper, we focus on the time dimension in the CoC because it reflects Cambodia’s policy goal that every woman should receive key packages of services across the pregnancy to postpartum continuum. Our study advances findings from previous continuum of care studies by uniquely considering whether the care components along the continuum were ‘optimal’ based on the recommendations of National Strategy of Reproductive and Sexual Health Cambodia [21]. These include four or more ANC visit with the first visit at the first trimester of pregnancy (optimal ANC), delivery assisted by SBA and at least 2 PNC visits within 41 days or six weeks of childbirth with the first PNC check is within 48 h after birth (optimal PNC).

Improving coverage along the continuum of care relies on a better understanding of factors contributing to gaps. Despite progress in MH indicators during the last decade in Cambodia, substantial coverage gaps remain [22] and more efforts are still needed to improve life-saving MH services. In this study, we aim (1) to investigate coverage along of the continuum of care, and (2) to identify determinants that are associated with mother’s continuation of service use along the continuum, and (3) to identify determinants of achieving a complete continuum of care.

## Methods

### Data source & study participants

This paper used data from the 2014 Cambodia Demographic Health Survey [2], a nationally representative household survey. The primary purpose of this survey is to provide detailed information on fertility and family planning; infant, child, adult and maternal mortality; maternal and child health; nutrition, and knowledge of HIV/AIDS and other sexually transmitted infections. The survey used a multi-stage clustered sampling design, which must be accounted for in statistical analyses.

Interviews were completed with 17,578 women of reproductive age (15–49). This study focused on women who had a live birth in the five years preceding the survey and were currently married (*n* = 5678), as questions on household decision-making were only asked to married women. Also, the prevalence of married women had a live birth was considerably high which was almost 95% of the total women in the study [2]. We further restricted our analysis to the women’s most recent live birth in the recall period as questions about ANC were only asked of the most recent birth. Detailed methodology and questionnaires used in the survey can be found in CDHS full report [2].

### Measurement

#### Dependent variables

The continuum of care for MH services, composed of three dummy variables: optimal ANC, SBA and optimal PNC, is the key outcome variable of this research. We defined optimal use for each of the three care components based on WHO and Cambodian Ministry of Health guidelines.

We defined optimal ANC as having received ANC at least four times during the pregnancy at either a health facility or at home, with the first ANC visit during the first trimester, based on WHO recommendations at the time the survey was conducted. The Cambodian Ministry of Health also encourages women to have their first ANC visits in their first trimester and count it as a crucial indicator in the National Strategies for Sexual and Reproductive Health [21, 23]. Thus, we treated it as a binary variable “optimal ANC” whereby code 1 is assigned to those who met the Ministry of Health recommendation and code 0 is assigned to those who did not meet this recommendation. Women reporting fewer than four visits or who did not begin ANC in the first trimester were considered to have not received optimal ANC.

SBA coverage referred to delivery assistance provided by health professional (a doctor, nurse, or midwife) either at home or health facility [24]. We employed it as a binary variable, where “skilled birth attendance” is assigned to those whose delivery was assisted by a doctor, nurse or midwife (code as 1) and “non-SBA” to those assisted by a traditional birth attendant, relative, friend, other non-health, or no one (code as 0), based on skilled attendant definitions used in the 2014 CDHS report [2].

We defined optimal PNC as checks on the woman’s health within 41 days or six weeks after childbirth. This reflects indicators used in the National Strategy for Reproductive and Sexual Health that women should receive at least 2 PNC checks between 48 h and six weeks post-partum and that the first check should occur in the first 48 h after delivery, regardless of delivery place. We coded 1 if women receive the first postnatal check up in the first 48 h after delivery and at least 2 PNC checks between 48 h and six weeks of post-partum and 0 if otherwise.

#### Independent variables

Predictors in the models include women’s socio-demographic (1), socio-economic (2) and cultural (3) factors which were found in the previous literatures to be associated with the use of maternal health services [25–30].

Socio-demographic factors are maternal age at the recent birth (< 20 years old, 20–34 years old, and ≥ 35 years old), women’s level of education (none, primary, and secondary or higher), and their husband’s level of education (none, primary, and secondary or higher). Cultural factors are any exposure to mass media (yes if they expose to one of the activities: reading newspaper or listening to radio or watching television at least once a week and no if they are not exposed to all of the three activities) and an index of decision-making power in the household (no power, some power and full power). Decision-making power in the household was based on the woman’s self-reported participation in a given decision related to three areas of decision-making in the household: determining own healthcare, making large household purchase and visiting family or relatives [2, 31]. We gave score 1 for each of the three areas of decision-making involving the woman or jointly with her husband and score 0 for each decision taken without the woman’s involvement (decision made by husband alone or other). We summed the score and assigned “no power” for index value 0, “some power in the decision-making” for index value 1 to 2, and “full power in decision-making” for index value 3.

Socio-economic factors included place of residence (urban or rural), employment categories of women and husband (did not work, non-agricultural work, or agricultural work), household wealth quintiles (poorest, poorer, middle, richer or richest) and health insurance coverage (yes or no).

We further considered the type of care received at previous stages in the continuum as predicting continuation to the next stage. For continuation to SBA, we examined four elements of ANC care which are the indicators of quality of optimal ANC care: whether the woman reported that (1) she was informed of signs of pregnancy complications, (2) had blood pressure measured, (3) had a urine sample taken and (4) had a blood sample taken. We classified women has having “received all different elements of ANC care” as “yes” if pregnancy women received all of the four services for their ANC check-up and as “no” if they were missing any one component. For continuation to PNC, we further considered delivery by caesarian section (yes or no) and whether the woman gave birth in a health facility (yes or no) as potential predictors of receiving PNC after receiving both ANC and SBA.

### Data analysis

We conducted a descriptive analysis of the characteristics of the study population and the level of coverage for each maternal health service along of the continuum (ANC, SBA and PNC), separately and together as a combined outcome. We examined the bivariate relationship between the independent variables and each dependent variable using chi-square tests and logistic regression was performed to estimate the associations with outcomes of interest while taking the other independent variables into account. Adjusted odds ratio (AOR) with 95% confidence intervals (CI) and p-values were calculated. The significance level was assigned at p ≤ 0.05. Sample weights and complex survey design were accounted for in both descriptive and logistic regression analyses. If missing value is less than 1%, that value will be automatically excluded from the analysis.

We conducted two logistic regression models. The first (A) contained of three sub-models (A-1, A-2 and A-3) which determined the extent of dropout along different points on the continuum of care and the second (B) contain a regression to determine factors associated with complete CoC.

**Fig. 1:**
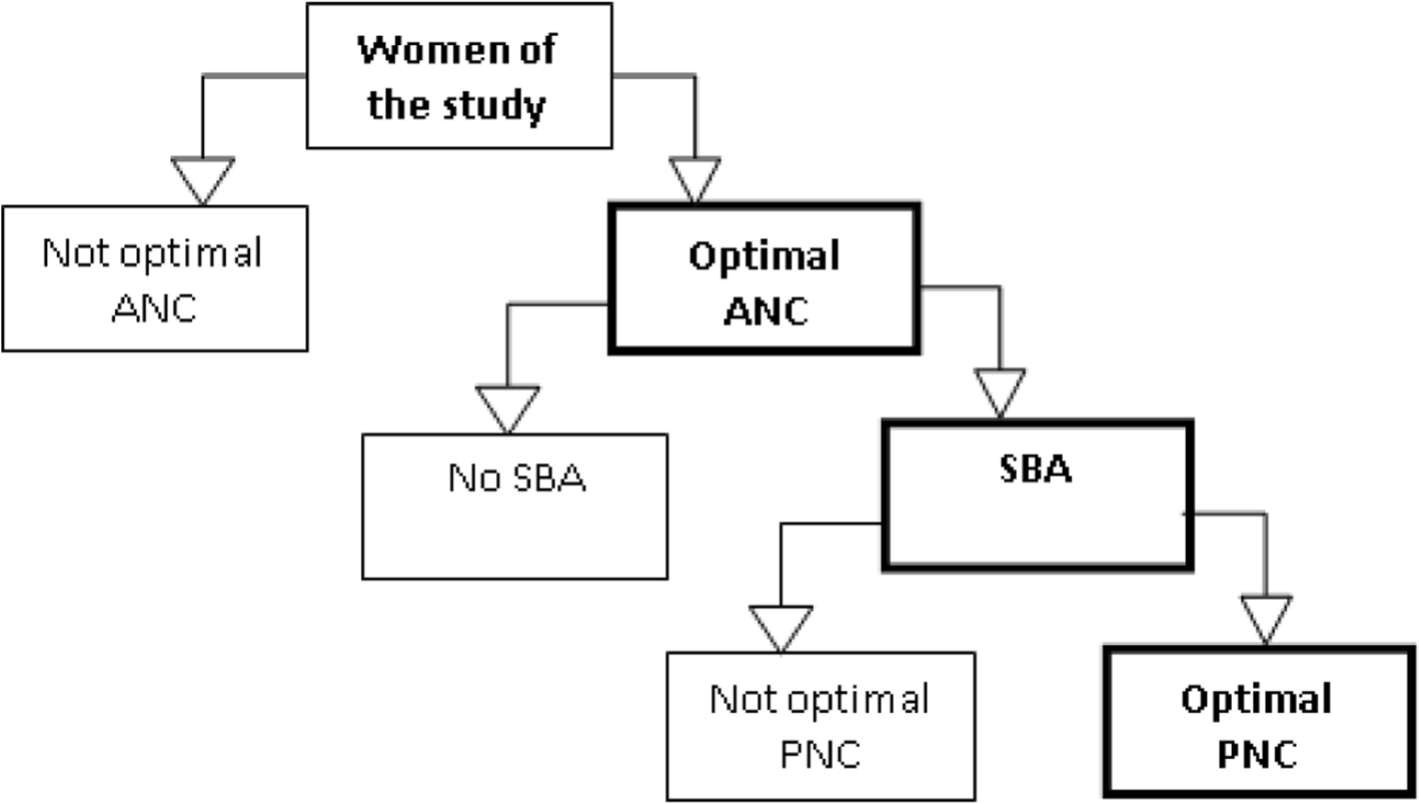
Model A illustrate the continuum of maternal health and the drop out from one service to another

**Model A** (Fig. 1):

**Model A-1** assessed the determinants of receiving optimal antenatal care (ANC) among married women with a live birth in the last five years (Code 1 if a woman received optimal ANC and O if woman do not receive optimal ANC).

**Model A-2** assessed the determinants of receiving skilled delivery (skilled birth attendance (SBA)) among the women who received optimal ANC care (Code 1 if for receiving optimal ANC and SBA, 0 for receiving optimal ANC but not SBA).

**Model A-3** assessed the determinants of receiving optimal postnatal care (PNC) among women who received optimal ANC and SBA (Code 1 for receiving optimal ANC, SBA and optimal PNC, 0 for receiving optimal ANC and SBA but not optimal PNC).

**Fig. 2:**
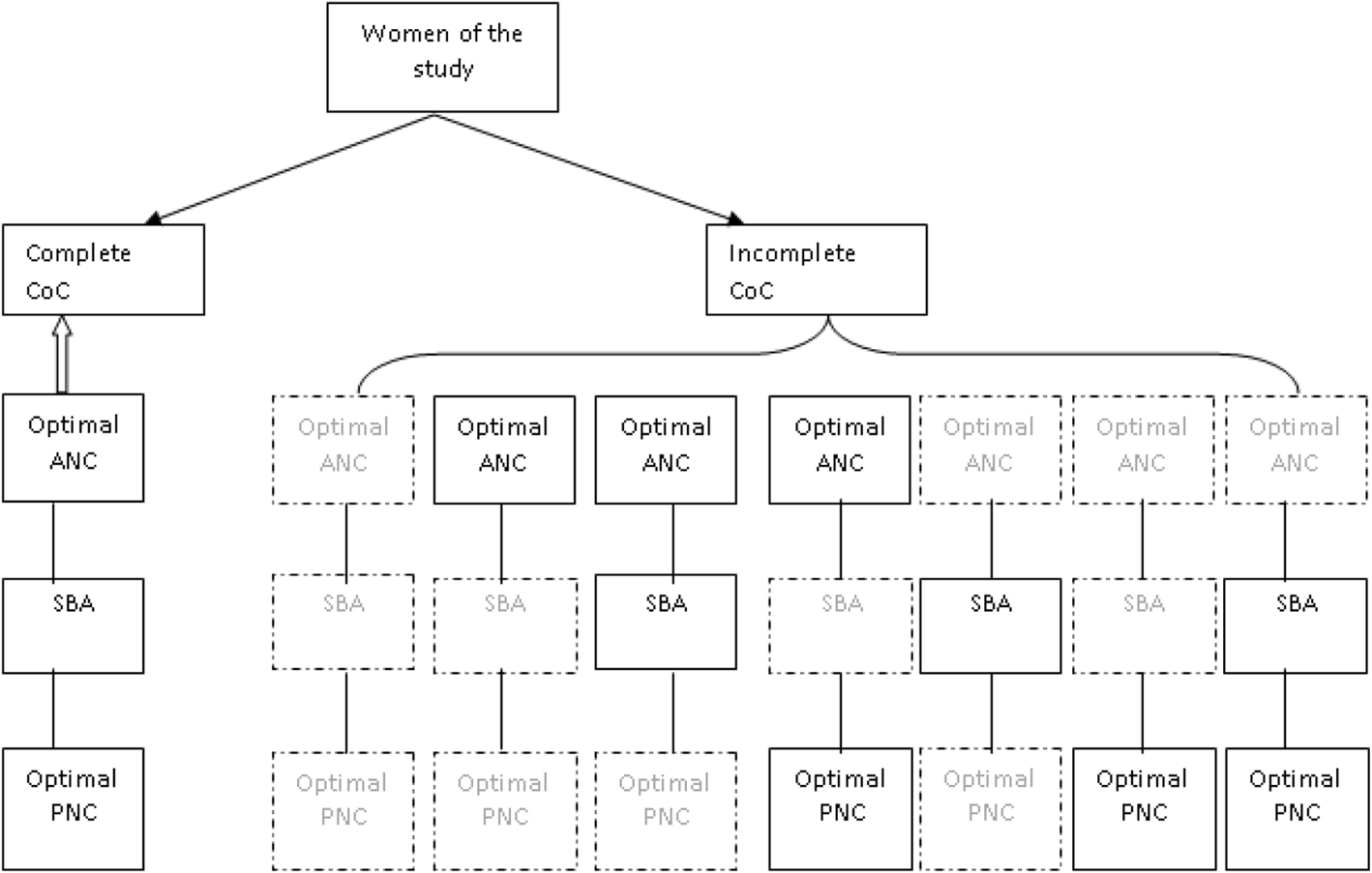
Model B illustrates the complete CoC VS incomplete CoC

**Model B** (Fig. 2) assessed the determinants of achieving complete CoC among all women. Code 1 is assigned to those women who achieved the complete CoC by receiving all of the three main outcomes and code 0 to those who received none or one or two of the three maternal health services.

## Results

### Characteristics of women in the study

The background characteristics of the married women who had at least one birth in the past five years interviewed are shown in Table 1. Most of the women included in the study had their most recent birth between the age of 20 and 34, had one or two children, completed more than primary level of education and were employed in non-agricultural sector. 86.0% of them were living in rural area and almost 65.0% has accessed to mass media, but only 17.0% of them was covered by health insurance. There is a homogenous proportion of each level of the wealth quintiles.

**Table 1:** Socio-demographic characteristics of married women who had at least one birth in the five years preceding the survey (weighted sample size = 5678 & unweight sample size = 5585)

Background characteristic	Weighted number (*n* = 5678)	Percentage (%)
**Mother’s age at birth**		
< 20	583	10.0
20–34	4525	80.0
35 +	570	10.0
**Birth order**		
1	1962	34.5
2	1772	31.2
3	947	16.7
4 or more	997	17.6
**Mother’s education**		
None	751	13.2
Primary	2971	52.3
Secondary or higher	1956	34.5
**Husband or partner’s level of education**		
None	530	9.0
Primary	2558	45.0
Secondary or higher	2586	46.0
Missing	4	0.1
**Mother’s employment**		
None	1401	25.0
Non-agricultural	2290	40.0
Agricultural	1986	35.0
Missing	1	0.01
**Husband’s employment**		
None	48.5	1.0
Non-agricultural	2903	51.0
Agricultural	2721	48.0
Missing	6	0.1
**Residence**		
Urban	818	14.0
Rural	4859	86.0
**Wealth quintile**		
Poorest	1303	23.0
Poorer	1156	20.0
Middle	1067	19.0
Richer	1020	18.0
Richest	1132	20.0
**Exposure to mass media**		
No	2068	36.4
Yes	3610	63.6
**Health insurance coverage**		
No	4688	82.6
Yes	990	17.4

### Overall use of maternal health services in Cambodia

Table 2 shows descriptive analysis results indicating that Cambodia has achieved high antenatal care coverage. Over 79.0% had the recommended four or more visits, and almost 80.0% of women in the study had their first ANC visit in the first trimester. Overall coverage of optimal ANC was nearly 70.0%.

**Table 2:** Number and Percentage of married women and their use of maternal health services

characteristics	Weighted Number (*n* total = 5678)	Percentage (%)
**Attend 4 + ANC visits (ANC ≥ 4)**		
No	1367	24.0
Yes	4310	76.0
**Attended first ANC at the first trimester**		
No	1175	21.0
Yes	4503	79.0
**Optimal ANC (ANC ≥ 4 and 1**^**st**^** trimester)**		
** No**	**1792**	**31.5**
** Yes**	**3886**	**68.5**
**Delivery by SBA**		
** Not Skilled Attendant**	**544**	**9.6**
** Skilled Attendant**	**5130**	**90.4**
** None**	**2**	**0.04**
** Missing**	**22**	**0.03**
**Woman with a postnatal checkup in the crucial first two days after birth**		
First 48 h	5033	88.6
More than 48 h	67	1.2
No PNC	578	10.2
**PNC ≥ 2 between 48 h and 6 weeks postpartum**		
PNC < 2	1082	19.0
PNC ≥ 2	4596	81.0
**PNC optimal (first PNC < 48 h and PNC visit > = 2 between 48 h and 6 weeks)**		
** No**	**1709**	**30.0**
** Yes**	**3969**	**70.0**

More than 90.0% of women delivered with a skilled birth attendant. Over 80.0% delivered at health facility, of which almost 70.0% of deliveries took place at public sector facilities and 15.0% at private sector facilities.

For postnatal care, 90.0% of women had a PNC check after delivery, regardless of their delivery place. Of which 88.0% had a PNC checkup in the first 48 h after birth and around 80.0% had at least two checks between 48 h and 6 weeks’ post-partum, as recommended by the National Strategy for Sexual and Reproductive Health. The proportion of women with optimal PNC was 70.0%.

### Pathways of maternal healthcare

The three main outcomes (optimal ANC, SBA and optimal PNC) created eight different combinations of maternal health service use from complete use of all three maternal health services to not receiving any of the three services (Table 3). The three services defined in the study are the continuation from (1) having optimal ANC (ANC ≥ 4 and first ANC at the first trimester), (2) having delivery attended by skilled birth attendant (doctor, nurse or midwife) and (3) having optimal PNC (PNC ≥ 2 between 48 h and 6 weeks’ post-partum and the first PNC in the first crucial two days after delivery).

**Table 3:** Percent distribution of women by different types of maternal health services, Cambodia 2014

Pathway	Optimal ANC (1) (≥ 4 and at the 1^st^ trimester)	SBA (2)	Optimal PNC (3) (≥ 2 and within 48 h after delivery)	%	*N*
1	-	-	-	4.0	214
2	+	-	-	2.0	106
3	+	+	-	16.0	906
**4**	** + **	** + **	** + **	**49.4**	**2807**
5	+	-	+	1.2	67
6	-	+	-	8.5	482
7	-	-	+	3.0	161
8	-	+	+	16.5	935

Less than half of women in the study (49.4%) received all three maternal health services along the continuum, shown in pathway 4. Notably, 4.0% of women did not receive any of the three maternal health services examined in the study (pathway 1). Three groups that attended only one of the three services (pathways 2, 6 and 7) accounted for 2.0%, 8.5% and 3.0% of women, respectively. Thirdly, the three groups that received only two of the three services (pathways 3, 5 and 8) accounted for 16.0%, 1.2% and 16.5% of women, respectively.

## Results from the regression analyses

### Factors associated with the use of optimal ANC among all women in the study

    Model I analyses factors associated with the use of optimal ANC care among all the women in the study (Table 4). The results show that the use of optimal ANC is significantly associated with maternal age at birth, birth order, woman’s level of education, husband’s level of education, power in household decision making, husband’s occupation, wealth quintile, exposure to mass media, and health insurance coverage.

**Table 4:** Result from multivariate logistic regression

Characteristics	Model A						Model B (*N* = 5678)	
	**Model A-1** **(N = 5678)**		**Model A-2** **(N = 3882)**		**Model A-3** **(N = 3710)**			
	**Optimal ANC**		**Optimal ANC and SBA**		**Optimal ANC, SBA and optimal PNC**		**Achieve complete CoC**	
	**AOR**	**95% CI**	**AOR**	**95% CI**	**AOR**	**95% CI**	**AOR**	**95% CI**
**Mother’s age at birth**								
< 20	1		1		1		1	
20–34	1.93***	1.48–2.50	1.85	0.89–3.83	1.14	0.80–1.61	1.68***	1.29–2.17
35 +	1.80***	1.28–2.53	0.77	0.33–1.80	1.07	0.65–1.74	1.47*	1.04–2.08
**Mother’s education**								
None	0.72**	0.57–0.90	0.64	0.36–1.15	0.97	0.66–1.41	0.72*	0.55–0.95
Primary	1		1		1		1	
Secondary or higher	1.28**	1.07–1.54	1.24	0.70–2.17	1.34*	1.05–1.70	1.36***	1.14–1.62
**Birth Order**								
1	1		1		1		1	
2	0.83	0.67–1.02	0.32***	0.19–0.55	0.95	0.75–1.22	0.80*	0.67–0.97
3	0.56***	0.44–0.72	0.24***	0.13–0.45	1.08	0.78–1.48	0.65***	0.52–0.82
4 or more	0.28***	0.22–0.37	0.21***	0.10–0.41	0.87	0.59–1.30	0.36***	0.28–0.46
**Husband’s level of education**								
None	0.97	0.75–1.26	1.12	0.62–2.03	1.17	0.80–1.72	1.07	0.82–1.41
Primary	1	1	1		1		1	
Secondary or higher	1.68***	1.41–2.00	1.64**	1.11–2.43	1.17	0.93–1.48	1.55***	1.30–1.85
**Decision making power in the household**								
No Power	0.76	0.37–1.54	2.71	0.28–25.68	0.30**	0.14–0.64	0.35**	0.16–0.74
Has some power	0.78*	0.61–0.99	0.79	0.42–1.46	0.86	0.62–1.17	0.79*	0.64–0.96
Full power	1		1		1		1	
**Exposure to mass media**								
No	1		1		1		1	
Yes	1.39***	1.17–1.65	1.45	0.91–2.29	1.15	0.93–1.44	1.37***	1.16–1.62
**Mother’s occupation**								
None	1		1		1		1	
Non-agricultural	1.19	0.94–1.50	1.45	0.79–2.69	1.08	0.81–1.43	1.18	0.96–1.44
Agricultural	0.99	0.76–1.31	0.83	0.48–1.46	1.07	0.80–1.43	1.01	0.79–1.28
**Husband’s occupation**								
None	5.22**	1.69–16.05	1.04	0.14–7.55	2.51	0.89–7.09	3.60***	1.64–7.85
Non-agricultural	1		1		1		1	
Agricultural	1.04	0.85–1.27	1.05	0.67–1.63	1.02	0.79–1.31	1.04	0.85–1.26
**Place of residence**								
Urban	0.88	0.64–1.21	1.78	0.67–4.71	1.74**	1.17–2.59	1.28	0.96–1.71
Rural	1		1		1		1	
**Wealth quintile**								
Poorest	1		1		1		1	
Poorer	1.08	0.85–1.36	1.13	0.60–2.13	1.05	0.77–1.43	1.11	0.89–1.40
Middle	1.33*	1.03–1.73	2.18*	1.03–4.61	1.46*	1.05–2.02	1.58***	1.20–2.06
Richer	1.79***	1.33–2.41	2.47*	1.02–5.98	1.35	0.96–1.90	1.80***	1.35–2.39
Richest	1.84**	1.21–2.81	3.57*	1.07–11.89	2.13**	1.19–3.80	2.36***	1.54–3.62
**Health insurance coverage**								
No	1		1		1		1	
Yes	1.22*	1.03–1.45	1.32	0.74–2.35	1.30	0.97–1.74	1.35**	1.12–1.63
**Received all different elements of ANC care**								
No			1		1			
Yes			1.63*	1.01–2.65	1.47***	1.19–1.83		
**Mode of delivery (C-section)**								
No					1			
Yes					2.12**	1.34–3.64		
**Delivery at health facility**								
Not health facility					1			
Health Facility					1.33	0.87–2.04		

(* *p* ≤ 0.05, ** *p* ≤ 0.01, *** *p* ≤ 0.001)

Women between 20–34 years old (AOR = 1.93, 95%CI = 1.48–2.50) and more than 35 years old (AOR = 1.80, 95%CI = 1.28–2.53) are more likely to use optimal ANC care than those below 20 years old. Women who have completed secondary school or higher education (AOR = 1.28, 95%CI = 1.07–1.54) were more likely to use optimal ANC than women who completed primary education. Women whose husband completed secondary school or higher education (AOR = 1.68, 95% CI = 1.41–2.00) and did not work (AOR = 5.22, 95% CI = 1.69–16.05) have greater odds of having optimal ANC care. Any exposure to mass media (reading newspaper, listening to radio or watching TV at least once a week) increased the odds of having optimal ANC care by 39.0% (AOR = 1.39, 95% CI = 1.17–1.65). Women who have some power (AOR = 0.78, 95% CI = 0.61–0.99) in the household decision-making were less likely to use optimal ANC care than women had full power in making decision in their household.

Women from wealthier households used optimal ANC care at higher rate than those women from less wealthier households. Women from the middle wealth households (AOR = 1.33, 95% CI = 1.03–1.73), richer households (AOR = 1.33, 95% CI = 1.33–2.41), and richest households (AOR = 1.84, 95% CI = 1.21–2.81) all had higher odds of optimal ANC compared with those from the poorest households.

Being covered by health insurance was more likely to use the optimal ANC services than those whose were not (AOR = 1.22, 95% CI = 1.03–1.45).

### Factors associated with the continuation of using SBA among those who have optimal ANC

Model II showed the factors predicting the maternal health service continuity of using skilled birth delivery (SBA) among women who have received optimal ANC care (Table 4).

Out of the women who have received optimal ANC care, women from the middle wealth households (AOR = 2.18, 95% CI = 1.03–4.61), richer households (AOR = 2.47, 95% CI = 1.02–5.98), and richest households (AOR = 3.57, 95% CI = 1.07–11.89) had higher odds of receiving both optimal ANC and SBA care compared with those from the poorest households. Women whose husband completed secondary or higher education (AOR = 1.64, 95%CI = 1.11–2.43) were more likely to continue using SBA after had used optimal ANC.

Received all different elements of ANC care increases the odds of continuity of care from optimal ANC service to SBA (AOR = 1.63, 95% CI = 1.01–2.65).

### Factors associated with the continuation of using optimal PNC among those who have both optimal ANC and SBA

Model III showed the predictive factors of the maternal health service continuity of using optimal PNC among those who have received both optimal ANC and SBA (Table 4). Having completed secondary or higher education increased the odds of having optimal PNC by 34.0% (AOR = 1.34, 95%CI = 1.05–1.70). Among the women who already received optimal ANC and SBA, those had no power (AOR = 0.30, 95%CI = 0.14–0.64) were significantly less likely to use optimal PNC than those women who has full power in making decision in their household.

Women living in an urban area (AOR = 1.74, 95% CI = 1.17–2.59) and from wealthier households continued using maternal health services and thus optimal PNC care compared to comparable women living in rural areas and from the poorest households. Women from the middle wealth households (AOR = 1.46, 95% CI = 1.05–2.02) and richest households (AOR = 2.13, 95% CI = 1.19–3.80) were more likely to use optimal PNC care compared to those who were from the poorest households.

Out of the women who have both optimal ANC and SBA, the odds of continued optimal maternal health care and thus optimal PNC was higher for women with the received all different elements of ANC care (AOR = 1.47, 95% CI = 1.19–1.83) and whose birth delivery was done through caesarian (AOR = 2.21, 95% CI = 1.34–3.64).

### Factors associated with achieving the complete continuum of maternal health care

Model B analyses the predictors of receiving a complete CoC (receiving optimal ANC + optimal SBA + optimal PNC) (Table 4) and the results showed that women between 20 to 34 years old (AOR = 1.43, 95% CI = 1.10–1.85) and over 35 years old (AOR = 1.47, 95%CI = 1.04–2.08) were more likely to complete the continuum than those who were younger than 20 years old. Also, women who had their second birth (AOR = 0.80, 95%CI = 0.67–0.97), third birth (AOR = 0.75, 95%CI = 0.60–0.95) and above (AOR = 0.47, 95%CI = 0.36–0.62) had lower odds of achieving the complete CoC than those who had their first birth. The completion of secondary or higher education (AOR = 1.30, 95% CI = 1.08–1.560 and her husband (AOR = 1.40, 95% CI = 1.17–1.68) was a significant predictor of a complete CoC. Exposure to any mass media also increased the odds of achieving the complete CoC. Women with no power (AOR = 0.39, 95% CI = 0.18–0.80) and some power (AOR = 0.79, 95%CI = 0.64–0.96) in household decision-making were less likely to complete the entire CoC compared to those who had full power to decide.

The odds of women achieving the complete CoC were higher among middle income (AOR = 1.58, 95%CI = 1.20–2.08), richer (AOR = 1.80, 95%CI = 1.35–2.39) and the richest households (AOR = 2.36, 95%CI = 1.54–3.62) compared to the poorest households. Women whose husband did not work were three times more likely to complete the entire CoC (AOR = 3.60, 95%CI = 1.64–7.85) compared to women whose husband performed agricultural work. Having health insurance coverage significantly increased the odds of completing the entire of CoC.

## Discussion

Ensuring optimal service use along the continuum of maternal health care is a critical part of Cambodia’s national strategy to improve the health of mothers and newborns. This study contributes important knowledge to understanding the gaps, where women are lost from one service to another, and why women are unable to achieve the complete CoC.

Our findings indicated that more women dropping out from the continuum of care between optimal ANC and delivery than between delivery and optimal post-delivery. Less than half of women in our study received optimal coverage of the full continuum of care. This reflects the progress of the government in the past years; however, extra efforts and effective fill-the-gap intervention from the government and relevant organizations are still needed to accelerate the level of optimal coverage through connecting the three services (optimal ANC, SBA and optimal PNC) together.

Women dropping out from one service to another due to some influential factors. Our finding that indicated the significant association between the use of optimal ANC with higher level of education, household wealth quintile and any exposure to mass media was consistent with previous studies which found that maternal education, husband education, household income, and media exposure were factors affecting ANC uptake in Pakistan [29], Uganda [32] and some developing countries [33].

Moreover, the finding that the higher levels of education of husband, and women from wealthier household positively associated with the continuity of using SBA among those had optimal ANC is in line with the result of an analysis of the Ethiopian Demographic and Health Survey which found that women whose husband completed at least secondary school were more likely to use skilled birth delivery assistant [34]. However, our study did not show a significant effect of women’s education on the continuity of having SBA among those had optimal ANC. Among married women who have both optimal ANC and SBA, over 60% were educated and came from at least medium income family. In this context, was wealth quintile became the most influential factor associated with the continuity of using SBA among those who have optimal ANC.

Received all different elements of ANC care is found to be a significant predictor of continuity of having SBA among those who had optimal ANC and continue to use optimal PNC among those who had optimal ANC and SBA. The elements of ANC care receiving during ANC visit make women better aware about their pregnancy condition and more likely to understand the importance of safe delivery and post-delivery care. A similar findings from a multi-country study of Africa indicated that the element of ANC care has a positive impact on delivery assistance and PNC use [35]. This demonstrate the importance of maintaining the quality of ANC care through enriching its content. WHO 2016 New ANC model also mentioned that “ANC should be delivered in term of both timing and content of each ANC contact” [36].

From the analyses, women’s decision-making power in the household was associated only with the use of optimal ANC care and the continuity of using of optimal PNC care for those had optimal ANC and SBA. This aligns with previous studies in Indonesia and Bangladesh which found that decision-making power had a positive effect on ANC and PNC coverage [37, 38]. In our study, decision-making power had no association with the continuity of using SBA after receipt of optimal ANC care. This is similar to another study in Indonesia in which women’s decision making power had no effect on the use of a skilled birth attendant [28], but different from the systematic review which found that women’s empowerment was positively associated with the use of maternal health service including skilled birth attendance in developing countries [39]. The difference in results from one to another study may be explained by the different measurement approaches to capturing women’s decision-making power [40]. One plausible explanation for why decision-making power is not a significant predictor on the continuity of using of SBA was that in delivery context, decision-making is not necessary and SBA is a usual event which a choice cannot be decided by only women and her family.

Our study also showed the predictors on the achievement of complete CoC. Decision-making power was also found in our study to be a significant predictor of achieving a complete CoC. This confirms a previous study in Nepal, using the same three outcomes, which found that women’s autonomy in decision-making had a positive effect on the use of all three maternal health services [41]. This suggests the new MH programs should refocus to empower women in making their own decision which in turn might accelerate the use of maternal health service. From our study, the cultural factors especially decision-making factors is an important determinant in achieving continuum of care. We have reflected the importance of social science research in pin-pointing the cultural factors that influence on barriers to access maternal health care. Future research should incorporate wider scope of social science into their study to better identify the barriers or gaps related to gender issue and health care utilization.

The educational levels of both women and husbands were significantly associated with achieving the complete continuum and thus offer a promising strategy for improving maternal health services in the country. The significant association between health insurance coverage and the complete achievement of CoC might due to the expansion of health-equity fund or mother health voucher schemes by the Ministry of Health Cambodia could also constitute a promising avenue. This reflects the coverage of social health protection program could encourage women to use services. Surprisingly, jobless husband was found to have greater odds for married women to achieve complete CoC. This could be the case since over 86.0% of the jobless group were from at least medium income family which they can afford the usage of services regardless of employment.

Consistent with many other studies, household wealth strongly predicted service use and achievement of the continuum of maternal care. A study in Gabon also found that maternal health service utilization was higher in women from wealthy households [42]. This implies that wealth can also be barrier to assess to care if the government has no strategies in removing financial barriers or financial hardship for women in using the services. Multi-sectoral actors and different attentions are needed to improve the continuity of care in Cambodia.

### Limitations

This study is subject to several limitations. The study was a cross-sectional study which we cannot use to make causal interpretations. The secondary data source used did not allow us to take the factors of maternal complications and need for care into analyses. Selection of the four indicators to define the complete elements of ANC care were based on data availability, and future studies would be strengthened by collecting data on and considering the role of quality of care received as predicting continuation to the next service. Due to skip patterns in the questionnaire, we were limited to examining only decision-making power among married women which the prevalence of married women giving birth was considerably high at around 95% in CDHS 2014 [2]. Because of the cross-sectional design of the study, all of the variables analyzed in the logistic regression especially decision-making power came from the time of the survey, the result can only provide statistical association and cannot show cause-effect relationship. Finally, the outcomes of our analyses relied on self-report, with the potential for misreporting of care received several years before the interview.

## Conclusions

Even with high coverage of each individual maternal health care service, Cambodia has achieved only a moderate proportion of women who complete the Continuum of Care from pregnancy to post-delivery with optimal care. This coverage would have been higher had only the count on the number of visits for ANC and PNC been considered, but our study further considered whether the visits were at the recommended time to help identify potential gaps of care. This is the ever first analysis examining optimal service use along the continuum and what factors should be taken into account or invested to improve the coverage of care. Policy makers should not only focus on the routine or common factors across all three services separately but also study the continuum in an integrated manner to identify how problems differ along the continuum. Social science research in Cambodia is limited; therefore, government should invest more in social science in order to widen the opportunities in identifying the influential factors of barriers accessing to health care especially on gender inequality. Future maternal health intervention should address this issue by consider including a component to empower women in decision-making and engage their spouse as well as other family member to understand the importance of using maternal health service as a continuum.

## Data Availability

Required permission was obtained from the DHS programme to access the data analysed for this study. All data and DHS-related materials used are available from the website: https://dhsprogram.com/.

## Abbreviations

MDG: Millennium Development Goal
SDG: Sustainable Development Goal
CoC: Continuum of Care
MH: Maternal Health
WHO: World Health Organization
ANC: Antenatal Care
SBA: Skilled Birth Attendant
PNC: Postnatal Care
HIV/AIDS: Human Immunodeficiency Virus infection and acquired immunodeficiency syndrome
CDHS: Cambodia Demographic Health Survey
AOR: Adjusted odds ratio
CI: Confidential Interval
C-section: Caesarian Section
